# Advancing healthcare through mobile collaboration: a survey of intelligent nursing robots research

**DOI:** 10.3389/fpubh.2024.1368805

**Published:** 2024-11-26

**Authors:** Boyuan Wang, Shanji Chen, Gexin Xiao

**Affiliations:** ^1^Beijing Xiaotangshan Hospital, Beijing, China; ^2^The First Affiliated Hospital of Hunan University of Medicine, Huaihua, China; ^3^Hunan Primary Digital Engineering Technology Research Center for Medical Prevention and Treatment, Huaihua, China; ^4^National Institute of Hospital Administration (NIHA), Beijing, China

**Keywords:** internet of things, mobile collaboration, nursing robots, artificial intelligence, clinical applications, review literature

## Abstract

Mobile collaborative intelligent nursing robots have gained significant attention in the healthcare sector as an innovative solution to address the challenges posed by the increasing aging population and limited medical resources. This article provides a comprehensive overview of the research advancements in this field, covering hospital care, home older adults care, and rehabilitation assistance. In hospital settings, these robots assist healthcare professionals in tasks such as patient monitoring, medication management, and bedside care. For home older adults care, they enhance the older adults sense of security and quality of life by offering daily life support and monitoring. In rehabilitation, these robots provide services such as physical rehabilitation training and social interaction to facilitate patient recovery. However, the development of intelligent nursing robots faces challenges in technology, ethics, law, and user acceptance. Future efforts should focus on improving robots’ perceptual and cognitive abilities, enhancing human-robot interaction, and conducting extensive clinical experiments for broader applications.

## 1 Introduction

With the rapid global aging population and increasing demand for patient care, healthcare resources are facing unprecedented challenges. For example, it is expected that from 2020 onwards, China will enter a serious aging stage, as the number of people over the age of 60 has reached 185 million, accounting for 13.9 percent of the total population according to China’s sixth national census. The United Nations has classified China as an “aging country,” and the rate of aging is accelerating. It is projected that the aging level will reach 17.17% by 2020. By 2050, China will become a super-aging country, with 30% of the population aged 60 and above (1), this indicates that China is facing a severe situation of population aging. Simultaneously, on a global scale, the trend toward an aging population is evident across all 195 regions (2). Japan is recognized as one of the world’s most rapidly aging societies and faces rising healthcare costs alongside a severe shortage of long-term care resources (3). Similarly, European countries such as Germany and Italy are experiencing significant increases in their older adults populations, which is placing pressure on their healthcare systems (4). Consequently, the escalating global demand for medical treatment, care, and rehabilitation, compounded by the relative shortage of medical personnel, is placing immense pressure on the capacity of health services (5).

With the rapid global aging population and increasing demand for patient care, healthcare resources are facing unprecedented challenges. Traditional nursing models are currently unable to meet the growing demand for care, indicating the need for innovative solutions. Various methods, such as telemedicine (6) and home-based care programs (7), have been proposed to address these challenges; however, these approaches often fall short due to issues such as limited accessibility and scalability, as well as concerns regarding privacy and security.

In this context, mobile nursing robots emerge as a promising solution. These robots aim to assist the older adults in independent living, mobility, risk monitoring, and dietary planning (8). Mobile collaborative nursing robots, as highly integrated artificial intelligence applications, primarily perform various specialized tasks in the nursing field, including vital sign monitoring, medication preparation, venous blood collection, suctioning, and throat swab sampling. In hospitals, their applications cover guiding, disinfection, patient transfer, and logistics. Additionally, in the field of home rehabilitation, mobile collaborative nursing robots are used for caring for older adults individuals at home, home hygiene care, health recovery, and psychological companionship, providing new impetus for the development of traditional nursing disciplines (9). The versatile nature of mobile collaborative nursing robots enables them to meet the nursing needs of different fields and populations, offering broader prospects for application in the medical care industry.

Given the advantages of mobile collaborative care robots in meeting diverse care needs, they represent a significant advance in the field and are gaining attention in many countries. At present, nursing robot technology in many developed countries has been at the forefront of world development. The development of China’s service robot industry has been listed as a key project of the “863” project and has attracted the attention of relevant departments and scientific research units (10). Although nursing robots are used in some medical institutions and scientific research experiments, there are still certain shortcomings in the popularization and widespread use of the entire field of nursing, mainly including technical limitations, high costs, stereotypes and social acceptance, legal and ethical issues (11). These problems largely interfere with the clinical application and efficiency release of nursing robots.

This paper primarily reviews the applications of different types of nursing robots in various contexts both domestically and internationally, including clinical professional nursing, auxiliary and support tasks, as well as the use of nursing robots in home-assisted care (see Tables 1, 2). Specifically, this study aims to address the following research question: ‘How can the integration of mobile collaborative nursing robots into clinical and home care environments improve the efficiency and quality of nursing services, and what are the major challenges and ethical considerations associated with their implementation?’ Through a comprehensive review of the literature, this study seeks to gain a deeper understanding of the current state of nursing robotics technology and identify potential strategies to optimize their application.

**Table 1:** Various nursing robots applied to different clinical tasks.

Application fields		Robot name	Key features	Limitations	Application challenge
Specialized Nursing tasks	Vital sign monitoring	Nurse Robot (NR) (28)	Uses Arduino Uno and multiple sensors. Employs NFC and IR for patient identification.	Possible sensor accuracy issues. Data transmission reliability concerns.	Integration with existing healthcare systems. Training healthcare providers.
		ANRD (29)	Manufactured using 3D printing technology. Monitors vital signs and assists with gait cycles.	Potential limitations in sensor accuracy. Possible issues with consistent connectivity.	Seamless integration into healthcare workflows. Training staff on its usage.
		Dr. Spot (31)	Equipped with infrared and RGB cameras for remote monitoring. Enables quick detection of abnormalities within 10 s.	Potential limitations in the accuracy of remote monitoring. Dependence on trained staff for operation.	Ensuring reliable and consistent remote monitoring. Training healthcare staff to operate the system effectively.
	Medication dispensing	Medication Delivery Robot (33)	Capable of autonomous navigation to retrieve medication. Simplifies medication retrieval through pick-and-place operation.	Potential errors in medication retrieval. Requires a well-categorized stockpile for efficiency.	Ensuring accurate medication selection and retrieval. Maintaining an organized stockpile system.
		APOTECAchemo (34)	More accurate than manual dispensing. Enhances safety and efficiency of medication preparation.	Potential system malfunctions. Initial setup and training requirements.	Ensuring reliable system performance. Training personnel to use the system effectively.
		WEINAS-VD 160 and WEINAS-PD 160 (35)	Achieves infusion dose accuracy near 95%. Enhances safety and accuracy with real-time drug mixing monitoring.	Potential for system errors. Dependence on advanced algorithms for accuracy.	Ensuring consistent system performance. Training users on advanced functionalities.
		MINA (Multi-Task Intelligent Nurse Assistant Robot) (36)	Assists with daily object retrieval using a robotic arm. Navigates accurately using an augmented reality interface.	Potential navigation errors or inaccuracies. Dependence on AR interface for waypoint setting.	Ensuring reliable and repeated task completion. Training staff to use the AR interface effectively.
		Dispensing Robot (37)	Improves efficiency and safety of intravenous medication dispensing. Reduces medication residues and dispensing errors.	Potential system errors or malfunctions. Initial setup and training requirements.	Ensuring consistent performance and accuracy. Training personnel to operate the system safely.
	Venous blood collection	MagicNurse (39)	Achieves a 94.3% puncture success rate. Less painful and preferred by patients for blood collection.	Potential variability in sample collection metrics. Requires initial setup and user training.	Ensuring consistent sample quality. Training staff to operate the robot effectively.
		Fully Automated Robot Venipuncture Device (40)	High success rates for venous access (87% for difficult, 97% for non-difficult). Reduces patient harm and decreases surgical time and costs.	Potential technical malfunctions. Dependence on accurate ultrasound imaging.	Ensuring reliability across different procedures. Training personnel on proper usage.
		Automatic Blood Collection Device (41)	Improves collection efficiency and user experience. Reduces risk of bloodborne infections and training costs.	Potential technical issues with ergonomics design. Dependence on user-friendly interface effectiveness.	Ensuring reliable and safe operation. Training users on the ergonomic design.
	Suction care	Smart Suction Nursing Robot (43)	Smooth and stable motion, easy to operate. Effectively performs suction tasks through simulated hand movements.	Potential mechanical failures or inaccuracies. Dependence on precise mechanical arm and hand coordination.	Ensuring consistent and reliable suction performance. Training personnel to use the advanced suction system.
		Fully Enclosed Suction Tube Flushing Device (44)	Adheres to bacteria-free operation standards. Removes adhered sputum, reducing VAP and minimizing waste.	Potential mechanical issues with pulse flushing. Dependence on precise control of flushing solution amounts.	Ensuring reliable and safe removal of sputum. Training on the use of the fully enclosed system.
		SPUTA VACCUMER (45)	Non-invasive phlegm and foreign substance clearance. Cost-effective and simple to use, even by non-professionals.	Potential limitations in effectiveness without tracheal intubation. Dependence on proper usage to ensure safety and efficacy.	Ensuring safe and effective use in various settings. Training users on correct operation procedures.
Basic Nursing Work	Hospital guidance	Intelligent Hospital Guidance Robots (48)	Reduce interpersonal contact and prevent virus spread. Provide real-time images and emergency contact services.	Potential technical issues with real-time image transmission. Dependence on reliable remote communication infrastructure.	Ensuring stable and secure real-time data transmission. Training users on emergency contact features.
		IWARD Mobile Service Robot (49–51)	Provides route guidance in complex hospital environments. Adjusts speed to match user’s pace for comfort.	Potential navigation issues in crowded areas. Dependence on plug-and-play functionality for ease of use.	Ensuring reliable navigation in dynamic settings. Training users on interacting with the robot.
		LIGHBOT (52)	Enhances ease and safety with adaptive speed based on user force. Improves navigation for visually impaired and older adults individuals.	Potential limitations in user force detection accuracy. Dependence on touch panel usability.	Ensuring reliable speed adaptation. Training users on touch panel operation.
		Vici Robot (53)	High-definition screen and camera for videoconferencing. Enables remote assistance without physical contact.	Potential videoconferencing technical issues. Dependence on stable internet connectivity.	Ensuring reliable videoconferencing service. Training users on remote operation.
	Disinfection and sterilization	Chlorine Dioxide Sterilization Robot (55)	Sprays disinfectants with a 99.8% *E. coli* clearance rate. Operates autonomously using IoT and gesture recognition.	Potential limitations in disinfection coverage. Dependence on accurate gesture recognition.	Ensuring thorough disinfection in all areas. Training personnel on autonomous operation.
		Automated UVC Light Mobile Robot (AUMR) (56)	Equipped with UVC light systems for sterilization. Operates automatically in various room types.	Potential UVC light safety concerns. Dependence on magnetic line sensors and fuzzy reasoning system.	Ensuring safe UVC light operation. Calibration and maintenance of sensors and systems.
		UVC Light Robot (57)	Efficiently disinfects rooms in 10 min. Reduces disease exposure risk and human error.	Potential UVC light safety concerns. Dependence on accurate positioning.	Ensuring safe and effective disinfection. Proper positioning and deployment in hospital rooms.
		LightStrike UV Disinfection Robot (58)	Uses pulsed UV with higher pathogen killing intensity. Disinfects a room in 20 min.	Potential safety concerns with pulsed UV. Dependence on consistent power and maintenance.	Ensuring consistent disinfection effectiveness. Regular maintenance and operational training.
		LD-UVC Robot (Omron) (59)	Automates UV disinfection of high contact points and rooms. Versatile for other applications like material handling and logistics.	Potential safety concerns with UV disinfection. Dependence on effective automation and navigation.	Ensuring safe and thorough disinfection. Training on diverse application capabilities.
		Gao Xian Intelligent Cleaning Robot (61)	Autonomously navigates and disinfects indoor spaces. Reduces need for protective gear, allowing safer and more efficient work for healthcare staff.	Potential navigation or disinfection inconsistencies. Dependence on accurate environmental understanding and task planning.	Ensuring reliable and precise movement planning. Training on the use of the robot for optimal efficiency.
	Patient transportation	OmniBed (63)	Converts manual stretchers into powered omnidirectional beds. Reduces manpower and offers safer, more ergonomic handling.	Potential technical issues with omnidirectional mobility. Dependence on proper attachment and operation.	Ensuring reliable and precise ‘parallel parking’. Training users on proper attachment and use.
		Robear (64)	Strong support and agile movements. Intelligent sensors for rapid perception and safe patient transfer.	Potential sensor malfunction or misperception. Dependence on accurate and responsive sensor technology.	Ensuring reliable and safe patient transfers. Training personnel on proper use and sensor interaction.
		Flexbed (65)	Autonomous navigation for safe and rapid transportation. Efficient obstacle avoidance and handling in crowded corridors.	Potential navigation errors or obstacle detection issues. Dependence on reliable collision avoidance strategies.	Ensuring consistent and safe navigation. Training on the use of autonomous features.
	Logistics transportation	TUGs (68)	Automates transportation of medications and supplies. Uses wireless network communication for elevator control and obstacle avoidance.	Potential wireless network connectivity issues. Dependence on accurate floor plan data.	Ensuring reliable obstacle avoidance and navigation. Training staff on wireless communication protocols.
		Aethon TUG (69)	Performs scheduled deliveries and tasks effectively. Transports a variety of items including surgical instruments and waste	Potential scheduling conflicts or delivery errors. Dependence on precise task scheduling and execution.	Ensuring consistent performance and delivery accuracy. Training on scheduling and task management.
		Zipline (70)	Unmanned aerial vehicle for transporting medical samples and supplies. Effective in remote areas like Ghana and Rwanda.	Potential flight path disruptions or weather-related delays. Dependence on robust communication systems.	Ensuring reliable and timely deliveries. Regulatory compliance and airspace management.
		Moxi (71)	Socially intelligent with navigation and obstacle avoidance. Helps gather supplies, distribute PPE, and deliver medications.	Potential limitations in social interaction and learning. Dependence on hospital workflow integration.	Ensuring seamless integration into hospital workflows. Training staff on social interaction and operational aspects.

**Table 2:** Various nursing robots applied to different home care tasks.

Application fields	Robot name	Key features	Limitations	Application challenge	Ethical consideration
Caring for older adults individuals at home	Care-O-Bot (74)	Retrieves and carries daily items. Assists in contacting healthcare personnel during emergencies.	Potential technical failures or reminders not being heeded. Dependence on accurate emergency detection.	Ensuring reliable emergency assistance and item retrieval. Training users on all available features.	Ensuring user privacy and dignity are maintained. Obtaining informed consent from users.
	Home-Based Remote Healthcare System (75)	Remote monitoring and automatic health data collection. Sends alerts in case of falls and enables remote control via mobile phones.	Potential false alerts or missed detections. Dependence on stable mobile network connectivity.	Ensuring reliable vital sign tracking and alert mechanisms. Training users on remote control functionalities.	Protecting the privacy of the older adults users. Ensuring informed consent for data collection and monitoring.
	Personal Robot 2 (PR2) (76)	Autonomously navigates and perceives the environment in 3D. Assists with tasks like walking the dog, folding clothes, and opening doors.	Potential navigation errors or task execution issues. Dependence on accurate environmental perception.	Ensuring reliable task execution and navigation. Training users on the robot’s capabilities.	Respecting the privacy and dignity of the older adults. Obtaining informed consent for the robot’s use in care settings.
	HomeMate (77)	Provides sociable and reliable assistance tailored for the older adults. Offers diverse services including infotainment, video chatting, gaming, medication reminders, and errand services.	Potential limitations in engaging the older adults effectively. Dependence on user acceptance and interaction.	Ensuring engaging and useful service scenarios. Training users on the various functionalities.	Ensuring the ethical use of personal data. Maintaining the dignity and autonomy of the older adults.
Home hygiene care	Adjustable Sit-to-Stand Assistive Device (79)	Adapts to individual body sizes and alleviates body weight. Performs hygiene tasks and monitors vital signs for safety.	Potential mechanical failures or discomfort during use. Dependence on accurate vital sign monitoring.	Ensuring reliable performance and safety during hygiene tasks. Training users on the adjustment and use of the device.	Respecting user privacy during intimate care tasks. Ensuring informed consent for vital sign monitoring.
	Medium-Sized Wearable Robot (80)	Moves along the human body to assist in cleaning limbs. Provides safe and efficient cleaning with 94% effectiveness.	Potential issues with direct human-robot contact. Dependence on proper fitting and mobility.	Ensuring safe and thorough cleaning. Training users on proper wear and use.	Ensuring privacy and dignity during intimate care. Obtaining informed consent for robotic assistance.
	Haksh-E (81)	Promotes good handwashing practices among children. Uses non-verbal expressions to enhance communication.	Potential limitations in maintaining children’s interest. Dependence on effective animation and interaction design.	Ensuring engaging and educational interaction. Training educators on the use of the robot.	Ensuring the content is age-appropriate. Obtaining parental consent for interaction.
	RABBIT (Robot-Assisted In-Bed Bathing System) (82)	Integrates multimodal perception and dual compliance for safe interaction. Uses RGB and thermal imaging to accurately segment skin areas for washing, rinsing, and drying.	Potential inaccuracies in skin area segmentation. Dependence on robust imaging technology.	Ensuring safe and comfortable bathing experiences. Training caregivers and users on the system’s operation.	Ensuring user privacy and dignity during bathing. Obtaining informed consent for the use of the system.
	Multidimensional Capacitive Sensing Technology (83)	Enables accurate and flexible perception and tracking of the human body. Supports daily living and hygiene needs, including activities like bathing.	Potential inaccuracies in sensing or tracking. Dependence on continuous technological refinement.	Ensuring reliable and accurate assistance. Training users on the capabilities and limits of the technology.	Ensuring user privacy and dignity during care. Obtaining informed consent for the use of sensing technology.
Health rehabilitation	PARR (Parallel Rehabilitation Robot) (85)	Features three rotational degrees of freedom around the ankle joint’s virtual fixed center. Includes a comprehensive information collection module to enhance human-robot interaction.	Potential alignment issues between the ankle joint and the virtual fixed center. Dependence on accurate information collection and processing.	Ensuring precise alignment during rehabilitation. Training users on the interaction and operation of the robot.	Ensuring patient privacy and data security. Obtaining informed consent for the use of the rehabilitation system.
	Soft Robot Ankle-Foot Orthosis (86)	Inexpensive, lightweight, and easy to wear. Provides gait assistance for rehabilitation in both clinical and daily life settings.	Potential limitations in custom fit for all users. Dependence on consistent and correct wear.	Ensuring effective gait assistance and comfort. Training patients on proper use and maintenance.	Ensuring patient safety and informed use. Obtaining informed consent for the use of the orthosis.
	i-Walk Smart Robot (87)	Integrates sensing, navigation, and user-robot interaction modules. Provides walking assistance, indoor and outdoor navigation, and health monitorin	Potential limitations for users with severe mobility impairments. Dependence on accurate sensing and navigation technologies.	Ensuring reliable support and assistance for users. Training users on the full range of features.	Ensuring user safety and privacy. Obtaining informed consent for health monitoring.
	Paro (88)	Mimics a baby seal to serve as a therapeutic tool. Enhances social interactions and improves mood in dementia patients.	May not be effective for all patients. Dependence on patient engagement and interaction	Ensuring appropriate use in care settings. Training staff on the therapeutic benefits and use	Ensuring the emotional well-being of patients. Obtaining informed consent for therapeutic use.
Psychological health companionship	Kabochanjiqr (90)	Features human-like characteristics and mimics human interaction. Improves cognitive functions and alleviates anxiety in older adults and Alzheimer’s patients.	May vary in effectiveness depending on individual patient responses. Dependence on sustained engagement and interaction.	Ensuring consistent emotional support and engagement. Training users and caregivers on its interactive capabilities.	Ensuring the emotional well-being of patients. Obtaining informed consent for the use of the robot.
	Lio (91)	Equipped with a multifunctional arm. Friendly design accepted by staff and patients.	Potential limitations in task execution precision. Dependence on autonomous operation reliability.	Ensuring reliable and safe assistance. Training staff and patients on its functionalities.	Ensuring the dignity and privacy of patients. Obtaining informed consent for robotic assistance.
	QTrobot (92)	Designed for autism support with interactive learning sessions. User-friendly setup enhances home education for children.	Effectiveness may vary among different children. Dependence on consistent and engaged use.	Ensuring engaging and effective learning sessions. Training parents on its use for tailored education.	Ensuring the well-being and progress of children. Obtaining informed consent for use in education.
	Pepper (93–95)	Designed for cognitive training and health monitoring. Reduces anxiety and loneliness, improving social participatio	Effectiveness may vary among different individuals. Dependence on continuous and engaged interaction.	Ensuring consistent and beneficial interaction. Training users on the robot’s functionalities.	Ensuring the emotional well-being of users. Obtaining informed consent for the use of the robot.
	TIAGo (96)	Aids individuals with motor and cognitive impairments. Suitable for both domestic and clinical use.	Effectiveness may vary depending on impairment type. Dependence on adaptable interaction capabilities.	Ensuring practical assistance in varied settings. Training users on the robot’s capabilities.	Ensuring the dignity and privacy of users. Obtaining informed consent for the use of the robot.

## 2 Research method

This study employs a systematic approach to conducting a literature review, aiming to provide a comprehensive examination of the research advancements in the field of mobile collaborative nursing robots. To ensure a thorough and systematic literature review, a rigorous search strategy was utilized to identify relevant studies. Our methodology encompasses the following key steps.

### 2.1 Literature search strategy

Databases: The search was conducted across several scientific literature databases, including PubMed, Scopus, Web of Science, Association for Computing Machinery database, Electronics Engineers database, Google Scholar, and IEEE Xplore.

### 2.2 Keywords

We utilized a set of keywords such as “nursing robots,” “intelligent nursing robots,” “robotic nursing care,” “rehabilitation robots,” “mobile collaborative,” “clinical applications,” “healthcare assistance,” “home care,” “quality of care,” “challenges,” and “ethical considerations.” The search was performed using various combinations of these terms to maximize relevance and coverage.

### 2.3 Selection criteria

Inclusion Criteria: The studies were selected based on the following criteria: (1) Direct focus on research concerning mobile collaborative nursing robots. (2) Description of nursing robots’ applications in medical, home care, or rehabilitation settings. (3) Discussion on the impact of nursing robots on the efficiency and quality of care. (4) Provision of detailed information regarding technical aspects, clinical applications, or studies on user acceptance and ethical considerations.

Exclusion Criteria: The criteria for exclusion included: (1) Literature unrelated to the main theme. (2) Studies that only discuss theory without empirical validation. (3) Conference abstracts, editorials, or review articles.

### 2.4 Data analysis

The selected studies were critically analyzed to extract key findings relevant to the research question. The analysis focused on identifying common themes, assessing the quality of evidence, and synthesizing results to draw meaningful conclusions.

### 2.5 Structure of the paper

Introduction: Provides background information on the study and clarifies the questions and objectives the study aims to address. Research Method: Offers a detailed explanation of the literature search strategy, criteria for study selection, and the data analysis process. Literature Review: Gives an overview of current nursing robot applications, compares different types of nursing robots, and discusses challenges and ethical considerations. Discussion: Synthesizes the findings and their implications, suggesting potential strategies for optimizing the application of nursing robots. Conclusion: Summarizes the key findings and suggests directions for future research. References: lists the works cited in the paper.

The chosen structure ensures a logical progression from the introduction of the topic to the detailed analysis of the literature, culminating in a comprehensive discussion and conclusion. This ensures that the findings are presented in a clear manner and supports the overall aim of the study.

## 3 Definition, development process, and characteristics of mobile collaborative nursing robots

Robots are virtual or mechanical objects designed to assist in human daily activities. The United States has been utilizing robots in industrial settings since the 1960s, and their introduction to healthcare in the 1980s had a positive impact on nursing activities (12). Various types of robotic technologies contribute to patient care, including assisting with patient mobility, medication administration, health assessments, physiological parameter monitoring, and providing companionship (13).

A proposed classification based on the characteristics of human-computer interaction modalities is of high value. The Human-Robot Interaction (HRI) taxonomy that has been proposed is partitioned into three distinct clusters, each focusing on different aspects of HRI. This taxonomy offers a more universal approach in contrast to existing frameworks that often lack broad applicability (14). The first cluster of the taxonomy enables the categorization of the overarching context in which the interaction takes place, covering all domains extensively. It takes into account both the area of application and the nature of human exposure during human-robot interactions (15). Once this broader context is defined, robot attributes such as its function, physical characteristics, and level of independence can be specifically identified and categorized. The second cluster delves into team dynamics, further subdividing into the human role, team composition, communication pathway, and spatial proximity (16). This hierarchical arrangement of the taxonomy serves a dual purpose: it allows for a top-down examination of existing interactions, moving from general settings to individual team nuances, and it also serves as a guide for tailoring and enhancing HRI designs in a bottom-up manner (17). As shown in Table 3, to enhance usability, the taxonomy is supplemented with visual aids that underscore the three cluster domains and their associated sub-categories, offering a clear template for detailing specific HRI scenarios.

**Table 3:** The human-robot interaction (HRI) taxonomy can be divided into the following three distinct taxonomic groups (14–17).

Groups	Specific dimensions	Substance
Interaction type taxonomy	Physical interaction	Involves interaction through physical movements, the use of tools, or physical means.
	Perceptual interaction	Including visual, auditory, or tactile sensing.
	Cognitive interaction	Such as decision-making, planning, and learning.
Interaction role taxonomy	Active role	Such as setting tasks, guiding the robot, or making decisions.
	Passive role	Such as receiving information or services provided by the robot.
	Collaborative role	Humans and robots work together in the interaction, sharing tasks and responsibilities.
Interaction ENVIRONMENT TAXONOMy	Structured environment	Such as factory production lines or laboratories.
	Semi-structured environment	Such as homes or offices
	Unstructured environment	Such as outdoor environments or disaster sites.

Social Assistive Robots (SARs) aim to support independent living, primarily focusing on providing intelligent assistance. SARs can be categorized into service robots and assistive robots. Service robots play a crucial role in offering assistance in daily life, including aspects such as diet, health monitoring, reminders, and safety (18). Mobile service robots, with significant potential, can support various daily living capabilities such as object, person, or target detection, cognitive training, and entertainment.

Mobile collaborative nursing robots refer to intelligent robots with mobility, sensing and cognitive capabilities, and collaborative working abilities (19). They are designed to provide high-quality nursing services in clinical and home environments, originating from the integration of intelligent service robot technology and the healthcare domain (20). As shown in Table 4, the advantages of mobile collaborative nursing robots over traditional nursing robots include:

**Table 4:** Overview of the advantages of mobile collaborative nursing robots.

Advantage description	Type of function	Application scenarios
Mobility	With autonomous navigation and mobility, adaptive complex.	Ability to move flexibly in different environments such as hospitals, nursing homes and homes (21).
Sensing and cognitive abilities	Using advanced artificial intelligence algorithms, various sensors such as cameras, liDAR and sound sensors are integrated.	Perceive and understand the surrounding environment, patient status and behavior, and accurately obtain the patient’s physiological information, location and behavior pattern (22).
Collaborative working abilities	Collaborate effectively with other robots or medical personnel.	In the operating room, it can work with surgical robots to provide precise care support (23). In the nursing team, can communicate with the medical staff without obstacles, and coordinate the nursing work (24).
High-quality nursing services	Including vital signs monitoring, drug management, rehabilitation training and psychological support (25).	To provide high quality, personalized, safe and reliable care services to improve the health and quality of life of patients (26).

Mobility: Mobile collaborative nursing robots possess autonomous navigation and mobility, allowing them to flexibly move in various environments such as hospitals, nursing homes, and homes, adapting to complex and dynamic scene requirements (21).

Sensing and Cognitive Abilities: These robots integrate various sensors (such as cameras, lidar, and sound sensors) and advanced artificial intelligence algorithms to perceive and understand the surrounding environment, patient status, and behaviors (22). This enables accurate acquisition of patient physiological information, location, and behavior patterns, facilitating personalized and precise nursing services.

Collaborative Working Abilities: Mobile collaborative nursing robots have the capability to collaborate with other robots or healthcare personnel effectively. For example, in the operating room, nursing robots can collaborate with surgical robots to provide precise nursing support (23). In nursing teams, these robots can communicate seamlessly with nurses and doctors, collectively completing nursing tasks (24).

High-Quality Nursing Services: Through interaction with patients and healthcare personnel, mobile collaborative nursing robots provide various nursing functions, including vital sign monitoring, medication management, rehabilitation training, and psychological support (25). Their goal is to deliver high-quality, personalized, safe, and reliable nursing services, improving patient health and quality of life (26).

Mobile collaborative nursing robots embody the integration of mobility, sensing, cognitive abilities, and collaborative functionalities. These intelligent systems are designed to deliver high-quality nursing services in both clinical and home settings, thereby addressing the continuously escalating demands of healthcare and promoting innovation within the medical field.

## 4 Application of mobile collaborative nursing robots in clinical settings

### 4.1 Overview

This section provides a comprehensive examination of the diverse roles played by mobile collaborative nursing robots within clinical settings. From specialized care tasks such as vital signs monitoring, medication dispensing, and venipuncture, to fundamental care duties like hospital navigation, disinfection, patient transport, and logistics management, advancements in dynamic mobile collaborative nursing robots offer innovative solutions aimed at enhancing patient care, boosting operational efficiency, and alleviating the workload on healthcare professionals. Each subsection delves into the specific applications and benefits of these robots, supported by examples drawn from current research and development in the field.

### 4.2 Purpose

The main purpose of this section is to illustrate how mobile collaborative nursing robots can contribute to modern healthcare practice. By detailing their capabilities and benefits, we aim to highlight the potential of these robots to transform care practice, improve patient outcomes, and address some of the pressing challenges facing the healthcare industry today, while also providing an analysis of the difficult challenges of mobile collaborative nursing robots in clinical practice.

### 4.3 Providing specialized nursing tasks

#### 4.3.1 Vital sign monitoring

Mobile collaborative nursing robots offer unique advantages in clinical settings by reducing close contact, lowering cross-infection risk, and minimizing PPE use. They are reliable and adaptable, providing rapid and accurate vital sign monitoring (27), presenting an innovative healthcare solution. Specifically, the Nurse Robot (NR) (28), a multifunctional nursing robot developed by the Egyptian College of Computing and Artificial Intelligence, utilizes an Arduino Uno board and multiple sensors. The system uses NFC to read patient labels and IR sensors to detect patients. It measures vital signs like heart rate, blood pressure, pulse, and blood oxygen saturation, transmitting data to physicians.

Mireles et al. (29) developed a new nursing mobile robot, ANRD. The structure and mechanical components of this robot device are manufactured using 3D printing technology. The instrument includes embedded electronic devices and sensors to understand the relative position between the robot and the patient. ANRD monitors vital signs such as electrocardiogram, blood oxygen saturation, skin temperature, and non-invasive arterial pressure, and assists with gait cycles for mobility-challenged individuals. It facilitates interactions between nursing robots, patients, and physicians, providing effective primary healthcare support.

The widespread impact of the COVID-19 pandemic has prompted the development of non-contact assessment methods for patients in hospital environments (30). To minimize face-to-face contact with potentially COVID-19-infected patients and preserve personal protective equipment, the research team at the Massachusetts Institute of Technology’s Department of Mechanical Engineering has created a mobile quadruped nursing robot system named “Dr. Spot” (31). The ‘Dr. Spot’ nursing robot, equipped with infrared and RGB cameras, facilitates remote vital sign monitoring including skin temperature, respiratory rate, and heart rate, via a tablet interface. Operated by trained staff, it allows for contactless operation, maintaining social distancing without the need to remove masks. This system enables quick detection of abnormalities within the first 10 s of interaction, significantly reducing contact between healthcare providers and patients, preventing disease spread, and conserving PPE. Its application is especially beneficial during the COVID-19 pandemic.

#### 4.3.2 Medication dispensing

With the rapid development of electronic information technology, increasingly intelligent software and devices are being applied to Pharmacy Intravenous Admixture Service (PIVAS). According to data released by the American College of Clinical Pharmacy (32), 0.3% of hospitals have introduced robotic systems for intravenous medication dispensing, leading to the avoidance of 5,420 medication errors, annual savings of $288,350, improved medication accuracy, enhanced safety, cost reduction, and time savings.

George and Megalingam (33) have designed and developed a prototype of a medication delivery robot capable of fetching the specified drug from a well-categorized stockpile through a straightforward pick-and-place operation. The process entails inputting the prescribed medication information, following which the robot autonomously navigates to the storage source and retrieves the required medicine.

A study by Iwamoto et al. (34) in Japan revealed that the robotic system APOTECAchemo for intravenous medication dispensing is more accurate and efficient than manual dispensing. This system significantly enhances the accuracy and safety of medication preparation. Wang et al. (35) utilizes the robotic systems WEINAS-VD 160 and WEINAS-PD 160 for medication dispensing. These robots integrate features like robotic vision, gravity sensing, advanced algorithms, and multi-axis arms, along with a comprehensive liquid transfer system. They achieve infusion dose accuracy near 95%, identify prescriptions, and monitor real-time drug mixing, enhancing safety and accuracy while reducing errors and occupational exposure risks.

Harish and Stephanie A. A. (36) have proposed a Multi-Task Intelligent Nurse Assistant Robot (MINA), designed to assist nurses with daily object retrieval, features a robotic arm on an omnidirectional mobile base. Using an augmented reality interface for setting waypoints, MINA can map and navigate to specified locations, such as supply cabinets, and complete grasp and retrieval tasks accurately and repeatedly.

Harbin Medical University Cancer Hospital utilizes the intelligent intravenous medication dispensing robot Dispensing Robot (37), improving the efficiency, convenience, and safety of intravenous infusion operations. This reduces the occurrence of medication residues and dispensing errors. During the medication dispensing process, the personnel are completely isolated from the medication, providing effective protection for operators and reducing injuries caused by medication preparation.

#### 4.3.3 Venous blood collection

Automatic blood collection nursing robots provide a positive patient experience, especially for those prone to fainting due to needles and blood. Moreover, they reduce healthcare workers’ needle stick injuries and contact infection (38), offering an innovative solution to improve nursing processes and enhance medical efficiency.

Wang et al. (39) evaluated the performance of the intelligent venous blood collection robot MagicNurse, demonstrating that the automatic blood collection robot outperformed manual collection in terms of specimen collection volume and pain. The robot achieved a 94.3% puncture success rate. Although there were statistically significant differences in 11 indicators of anticoagulant blood samples collected by the robot versus manual collection, these did not impact clinical diagnosis or prognosis. The robot’s blood collection method is less painful, well-received by patients, and suitable for clinical anticoagulant sampling.

Leipheimer et al. (40) developed a fully automated robot venipuncture device. Combining ultrasound imaging and micro-robot technology, the success rates for difficult and non-difficult venous access blood collection were 87 and 97%, respectively. This device improves venous access success rates, reduces patient harm, and decreases surgical time and institutional costs. Its applications include venous catheter insertion, central venous access, dialysis, and arterial catheter placement.

Furthermore, Chen et al. (41) designed an automatic blood collection device based on ergonomics and user journeys. This device aims to improve collection efficiency, reduce the risk of bloodborne disease infections caused by needle injuries to medical personnel, alleviate the workload and training costs for healthcare workers, and enhance the user experience.

#### 4.3.4 Suction care

Remote-controlled or automated robots can replace nursing staff, effectively reducing close contact between healthcare workers and infected patients, as well as minimizing exposure to high concentrations of droplets and aerosols in the air (42). This, in turn, lowers the risk of infection, alleviates the psychological burden and workload of healthcare workers, which is particularly crucial during the COVID-19 pandemic.

Tan et al. (43) developed a smart suction nursing robots that is stable in motion, easy to operate, and ensures a smooth process. By simulating the suction process with mechanical arms and hands, key parameters and procedural data were derived from clinical practices, informing the robot’s motion unit design. The robot replicates suction actions using thumb, index finger, and palm joints to grip the suction tube, mimicking wrist and elbow movements through servo and arm structures. Its feasibility was confirmed using an advanced suction training model. The robot performs smoothly, executing tasks like gripping, feeding, retracting, and rotating the tube effectively.

Peng et al. (44) designed a fully enclosed suction tube flushing device. This nursing robot features an integrated design with fully enclosed suction, humidification, and flushing materials, adhering to bacteria-free operation standards. The syringe’s pulse flushing removes adhered sputum, reducing ventilator-associated pneumonia (VAP). Usage controls the flushing solution amount, ensuring patient safety and minimizing waste. Additionally, Ishikita (45) from Japan invented a 3D-printable device called SPUTA VACCUMER, which can clear phlegm and foreign substances non-invasively without tracheal intubation, it is characterized by minimal invasiveness, simplicity, and cost-effectiveness. Manufacturable via 3D printing, its plastic injection model gained Japanese drug approval in Amritha Ashok et al. (46) and is now used clinically. Operable by non-professionals, it is also suitable for use in space to enhance astronauts’ quality of life.

### 4.4 Basic nursing work in the hospital

#### 4.4.1 Hospital guidance

Intelligent hospital guidance robots are poised to play a crucial role in the healthcare sector. They offer accurate navigation, enhance operational efficiency (47), reduce the risk of cross-infection, and provide personalized services for patients.

During the Global COVID-19 Pandemic, intelligent hospital guidance robots have been jointly developed by institutions such as Siao et al. (48) to reduce interpersonal contact during the pandemic, prevent the spread of the virus, and assist medical staff in patient guidance and communication services. These robots offer emergency contact information for users to seek assistance remotely, and provide real-time images and control permissions to security personnel for immediate situational awareness and response. Today, one common issue encountered in large hospital buildings is that patients struggle to navigate within the hospital. The IWARD project (49–51), funded by the European Union, investigates the utilization of mobile service robots to assist hospital staff in their daily tasks, offering route guidance services to patients and visitors within hospital facilities. This robot guides patients to the X-ray room or through complex, dynamic, and crowded hospital environments, adjusting its speed to match the user’s pace for comfort, and incorporates plug-and-play functionality.

Tobita et al. (52) have focused on providing efficient guidance support to visually impaired individuals and the older adults within large hospital environments, culminating in the successful development of a novel guidance robot, LIGHBOT. This robot uses a touch panel for destination setting and adapts its speed based on the user’s applied force, enhancing ease, safety, and user confidence. LIGHBOT, known for its applicability and practicality, significantly improves navigation for visually impaired and older adults individuals in healthcare facilities. The Vici robot (53), for example, developed by InTouch Health, has a high-definition screen and camera that enable doctors and nurses to assist patients via videoconferencing services, without physical contact.

#### 4.4.2 Disinfection and sterilization

The high infectivity and severity of the novel coronavirus (COVID-19) in 2019 underscored the necessity of hospital space disinfection technology and preventing human exposure to pathogenic environments (54).

Robot disinfection generally falls into two categories: ultraviolet light irradiation and chemical disinfection. Zhao et al. (55) from the Department of Mechanical Engineering at National Taiwan University have researched and developed a novel chlorine dioxide (ClO2) sterilization technology robot to reduce bacteria and viruses in the air and on surfaces. An intelligent disinfection robot, designed based on experimental results and hospital needs, sprays disinfectants in operating rooms and wards. Using IoT and gesture recognition, it operates autonomously with a 99.8% *E. coli* clearance rate.

Rusdinar et al. (56) developed a mobile sterilization robot equipped with UVC light systems at the top and bottom to emit UVC light. The Automated UVC Light Mobile Robot (AUMR) operates automatically using magnetic line sensors and a fuzzy reasoning system. Experiments show excellent sterilization performance in various room types, including positive and negative pressure environments.

UVD Robotics (57), a company based in Denmark, has introduced clinically tested and verified mobile UVC light robots into hospitals to efficiently disinfect rooms. The robot positions itself in the hospital room and approaches critical objects during the 10-min disinfection process, eliminating disease exposure risk to staff and reducing human error. Xenex, a U.S. company, produces the LightStrike UV disinfection robot (58), which uses pulsed UV (200–315 nm) with 4,300 times greater pathogen killing intensity than traditional UVC (254 nm) sources, disinfecting a room in 20 min. Deployed in over 400 hospitals globally, these robots enhance environmental hygiene practices.

Omron introduced the LD-UVC robot specifically to fight the COVID-19 pandemic, automating the UV disinfection process safely and wisely (59). This robot is used for effective disinfection of high contact points and rooms and is suitable for healthcare, hospitality, assisted living and commercial buildings. Due to its OMRON LD base, it can be used for various other applications such as material handling, catering, and other logistical needs.

During the COVID-19 pandemic, many AI technology companies in China continue to develop and fine-tune disinfection robots (60), donating them to hospitals in Hubei Province and other severely affected regions nationwide. Examples include the Titanium Intelligent Sterilization Robot, “Chuang Chuang” Sterilization Robot, and the Gao Xian Intelligent Cleaning Robot (61). These intelligent sterilization robots autonomously navigate and disinfect indoor spaces, planning movements precisely. They understand their environment and tasks, reducing the need for protective gear and allowing healthcare workers to focus on high-value tasks more safely and efficiently.

#### 4.4.3 Patient transportation

Patient transportation in hospitals faces numerous challenges, including limited manpower, work-related injuries, and inefficient traditional stretcher methods (62). Patient transportation nursing robots can autonomously perform patient transfer tasks, reducing the need for manual operations. They provide patients with safer, more comfortable, and efficient transfer experiences.

Guo et al. (63) have developed a novel electric robot bed mover called OmniBed, featuring omnidirectional mobility, this device attaches to a manual hospital stretcher, converting it into a powered omnidirectional bed (OmniBed) for single person uses. The OmniBed reduces manpower, decreases back muscle activity, and offers more ergonomic, efficient, and safer handling compared to traditional beds, with precise ‘parallel parking’ in small spaces.

To meet diverse nursing transportation requirements in various scenarios, Japan and the United States have jointly developed humanoid dual-arm patient transfer robots like Robear (64). These robots boast strong support and agile movements in addition to intelligent sensors for rapid perception of the external environment, assisting nurses in easily and safely transferring patients in different scenarios.

Wang et al. (65) have developed a new intelligent robot bed called Flexbed, equipped with autonomous navigation capabilities for rapid and safe transportation of critically ill neurosurgery patients without changing the bed. Compared to traditional hospital beds, the Flexbed is more efficient and safe during transportation. It avoids obstacles using collision avoidance strategies and moves at maximum speed when clear, navigating crowded corridors and overcoming mobility obstacles.

#### 4.4.4 Logistics transportation

Logistics transportation nursing robots can execute the transportation tasks of supplies and medications within medical institutions, including logistics distribution from warehouses to wards, operating rooms, and other locations (66). They can quickly deliver urgently needed supplies in emergency situations. With features such as automated transportation, efficiency, reliability, reduced risk of cross-infection, and conservation of human resources, these robots provide medical institutions with safe, efficient, and reliable material transportation solutions (67).

At the University of California, San Francisco Medical Center, an introduction of 25 intelligent transport robots called TUGs (68) assists medical staff in transporting medications, goods, laboratory specimens, and food. These robots automatically navigate through the hospital’s stored floor plan, avoiding obstacles at all times. They utilize wireless network communication to control elevator door opening and closing applications. Currently, TUGs are in use in multiple hospitals, with excellent feedback on their effectiveness. Another example of a hospital service robot is the “Aethon TUG (69),” which transports surgical instruments, medications, food and beverages, linens, and waste. The robot can perform scheduled deliveries or required tasks with outstanding performance.

In Ghana and Rwanda, the “Zipline (70)” unmanned aerial vehicle is used to transport coronavirus samples, blood products, and medications. Moxi uses a freight mobile base for navigation and obstacle avoidance, with a compliant arm and hand for manipulation. It is socially intelligent, learns from humans, and adapts to hospital workflows. Moxi has been piloted to gather patient supplies, distribute PPE, and deliver medications, helping staff spend more time with patients and provide efficient care support (71).

## 5 Application of nursing robot in home rehabilitation field

### 5.1 Overview

This section will discuss the application of mobile collaborative nursing robots in the field of home rehabilitation, including home care for the older adults, home health care, physical rehabilitation and psychological companionship. By introducing a variety of nursing robot systems and technologies developed in different countries and regions, we show how they support multiple aspects of patients’ daily life through intelligent functions, from providing social companionship to performing personal hygiene and care tasks to assisting in health rehabilitation training.

### 5.2 Purpose

This section aims to reveal how mobile collaborative nursing robots can improve the quality of care in home rehabilitation Settings and improve the lives of older people and those with special needs. Through the study of existing technologies and their practical application cases, it shows how these innovative solutions meet the growing needs of home care, and provides inspiration for future research and development directions.

### 5.3 Caring for older adults individuals at home

Homecare nursing robots possess intelligent technologies and features such as facial recognition, voice recognition, autonomous navigation, etc. (72). They offer advantages such as daily care provision, health condition monitoring, safety assurance, and social companionship (73).

Lorenz et al. (74) has developed a social companion robot for older adults care named Care-O-Bot. This robot can retrieve and carry common daily items, assist in contacting healthcare personnel during emergency situations (falls, etc.), provide support for daily care functions, remind older adults individuals to eat, and help them fetch items.

In China, Zhou et al. (75) has developed a new home-based remote healthcare system for the older adults based on mobile robots. This nursing system, with remote monitoring and automatic health data collection, constantly tracks the vital signs and activity of the older adults, sending alerts in case of falls. It also enables remote control of the robot via mobile phones.

Personal Robot 2 (PR2) (76) is a multifunctional artificial-assistance mobile nursing robot designed to provide independent living and interaction for humans, especially the older adults. PR2 autonomously navigates by perceiving the environment in 3D, assisting with tasks like walking the dog, folding clothes, and opening doors, providing home care for the older adults.

The robot “HomeMate” represents an innovative attempt based on its sociability and reliability (77), realized through extensive user studies. Older adults individuals cared for by senior welfare centers are chosen as the target group, for whom five service scenarios are designed: infotainment, video chatting, gaming, medication reminders, and, notably, errand services.

### 5.4 Home hygiene care

Hygiene care nursing robots typically have user-friendly designs, including soft materials, smooth movements, etc., to provide a comfortable experience (78). In China, Fu et al. (79) have designed an adjustable sit-to-stand assistive device for personal hygiene nursing robots. This device adapts to individual body sizes, alleviates body weight, and performs hygiene tasks like washing hair and drying the body. It includes vital sign monitoring for safety during bathing and features water and energy efficiency, compact design, and high comfort.

Liu et al. (80) have developed a medium-sized wearable robot that can move along the human body, assisting in cleaning the surface of limbs. This solution addresses direct human-robot contact challenges through wearable design and flexible mobility, providing a safe and efficient way to clean patients’ body surfaces with a 94% effectiveness, improving hygiene, comfort, and reducing caregiver workload.

The robot Haksh-E (81) is designed to promote good handwashing practices among children. It is engineered with two degrees of freedom and physically resembles an anthropomorphic soap dispenser. Its face features an animated mouth, human-like eyes with large irises, and eyebrows that enhance verbal communication through non-verbal expressions. This design is based on prior research in child-robot interaction scenarios.

RABBIT (82) is an innovative robot-assisted in-bed bathing system designed to meet the growing demand for assistive technology in personal hygiene tasks. It integrates multimodal perception and dual compliance for safe and comfortable physical human-robot interaction. RABBIT uses RGB and thermal imaging to accurately segment dry, soapy, and wet skin areas, performing washing, rinsing, and drying tasks according to professional nursing practices.

Erickson et al. (83) have developed a multidimensional capacitive sensing technology, offering a promising method for robots to perceive and track the human body in assistive tasks requiring human-robot interaction. This technology equips nursing robots with accurate and flexible capabilities to support the daily living and hygiene needs of disabled individuals, including activities like bathing. It shows promise and potential for future robot-assisted care.

### 5.5 Health rehabilitation

Health rehabilitation robots have achieved certain results in active training, flexible control, and rehabilitation assessment (84). Zhang et al. (85) has developed a parallel rehabilitation robot, PARR, based on end effector, featuring three rotational degrees of freedom around the virtual fixed center of the ankle joint. During rehabilitation, the ankle joint’s center should align with the virtual fixed center. The system includes a comprehensive information collection module to enhance human-robot interaction among the robot, patient, and doctor, making it widely applicable for ankle rehabilitation.

Kwon et al. (86) has proposed a soft robot ankle-foot orthosis for post-stroke patients. This orthosis is inexpensive, lightweight, easy to wear, and provides gait assistance for rehabilitation not only in clinics but also in daily life. It improves the walking propulsion force and prevents foot drop in patients, benefiting their gait and overall rehabilitation.

Moustris et al. (87) have developed the i-Walk smart robot, a comprehensive system integrating sensing, navigation, and user-robot interaction modules. It combines user-adaptive motion control, dynamic environment navigation, and cognitive assistance, suitable for individuals with mild to moderate mobility impairments. It provides stable body posture support, walking assistance, indoor and outdoor navigation, and health monitoring.

Paro, designed to mimic a baby seal, serves as a therapeutic tool to reduce anxiety and loneliness in dementia patients (88). Its interactive capabilities enhance social interactions and mood, improving patients’ behavioral outcomes. Used in care facilities and homes, Paro provides emotional support.

### 5.6 Psychological health companionship

Psychological companion nursing robots play an important role in providing emotional support and psychological counseling (89). Ke et al. (90) developed the humanoid social robot Kabochanjiqr, featuring human-like characteristics. Through interactive capabilities, mimics human interaction, meeting patients’ emotional needs. It improves cognitive functions in older adults women living alone and alleviates anxiety in Alzheimer’s patients.

Lio is a mobile robot platform equipped with a multifunctional arm, specifically designed for human-robot interaction and personal care assistant tasks. Its friendly appearance has led to widespread acceptance among healthcare staff and patients (91). This robot has already been deployed in several healthcare facilities, where it operates autonomously, assisting staff and patients on a daily basis.

QTrobot is an innovative social robot specifically designed to support the education and development of autistic children (92). QTrobot offers interactive and consistent learning sessions focused on speech, social–emotional skills, and cognitive development, making home education fun and effective. Its user-friendly setup empowers parents to provide tailored education, fostering children’s growth.

Pepper (93–95), the robot, is a long-term semi-humanoid autonomous synthetic aperture radar developed by Softbank Robotics, originating from Japan and France. This robot is designed for cognitive training, health monitoring, companionship, and more, this robot aids various groups including Alzheimer’s patients, older adults stroke survivors, those with depression, and healthy older adults. It effectively reduces anxiety, loneliness, and improves social participation. TIAGo robot can conveniently aid individuals with disabilities (96), including those with motor and cognitive impairments in both domestic and clinical settings.

## 6 Challenges and ethical issues in the application of nursing robots

With the significant growth of the world’s older adults population, the care of older adults and disabled individuals has become a focal point of societal and governmental concern. Mobile collaborative nursing robots play a crucial role in promoting social care services, contributing to the enhancement of patient and older adults care quality, and alleviating the burden on healthcare personnel (97). However, the development and application of nursing robots still face challenges and ethical issues. Based on our review, these issues are discussed in detail below.

The challenges are reviewed below: (1) Technological Challenges: Mobile collaborative nursing robots require high levels of intelligence and autonomous navigation capabilities to adapt to complex and dynamic clinical environments. This involves technical challenges such as perception and understanding of the environment, path planning, indoor navigation, maneuvering, telecommunications, and the integration of robots with existing hospital technology (98). (2) Safety Challenges: Nursing robots closely interact with patients and healthcare professionals, necessitating the assurance of their safety. Existing safety challenges include multiple personal safety risks such as uncontrolled systems, potentially harmful physical contact, falls and collisions (99), so robots need safety sensors and control systems to prevent harm to patients or others. (3) Human-Robot Interaction Challenges: Human-robot interaction is a critical aspect of nursing robot applications. Robots need to possess effective speech recognition, emotional understanding, and expressive capabilities (100) to facilitate communication and collaboration with patients and healthcare professionals.

Upon review, existing ethical issues include: (1) Privacy protection: providing nursing services may involve the acquisition and processing of patients’ personal privacy information. The uncertainty of data protection requirements creates privacy data security issues for users, especially for vulnerable robot users (101). Therefore, protecting patient privacy becomes a significant ethical issue, requiring the formulation of corresponding privacy protection policies and measures. (2) Ethical Decision-Making: In some cases, nursing robots may need to make decisions regarding patient care and treatment. This raises ethical decision-making issues (102), ensuring that robots adhere to ethical guidelines and make correct decisions in the best interest of the patient. (3) Responsibility and Accountability Issues: The interaction between nursing robots, patients, and healthcare professionals necessitates a clear delineation of responsibilities and accountabilities between robots and humans (103). This includes issues such as behavioral norms for robots, accountability, and legal responsibilities (104).

Based on a comprehensive review of the current challenges and ethical issues in the development and application of nursing robots, we believe that addressing the challenges and ethical issues in the application of nursing robots requires a multifaceted approach, including technological advances, legal regulations, and the establishment of ethical codes. To be specific: (1) Technological Innovation: Continuous technological innovation is essential to enhance the perception, cognition, and decision-making abilities of mobile collaborative nursing robots (105). Introducing advanced sensing technologies, artificial intelligence algorithms, and autonomous navigation systems can improve the adaptability and safety of robots in complex environments. (2) Legal Regulations: Establishing relevant laws and regulations to standardize the design, development, and use of nursing robots. This includes safety certifications for robots, privacy protection measures, and regulations on ethical decision-making (106). (3) Ethical Guidelines: Formulating ethical guidelines and moral principles to clarify the principles and ethical requirements of nursing robots. This involves provisions for protecting patient privacy, ethical decision-making principles, and regulations for accountability (107).

Mobile collaborative nursing robots have immense potential in application to enhance care efficiency, alleviate healthcare resource pressures, and improve patient care experiences. However, it is imperative to fully consider technological challenges and ethical issues, promoting the sustainable development of nursing robots through technological innovation and institutional development to achieve better care services and societal well-being.

## 7 Summary of the future development direction of nursing robots

Mobile collaborative nursing robots, as an innovative solution, have vast development prospects. With continuous technological advancements and increasing medical demands, the future development of mobile collaborative nursing robots will progress in the following directions:

(1) Enhancement of Intelligence and autonomy: future mobile collaborative nursing robots will become more intelligent, possessing advanced perception, cognition, and decision-making capabilities. Through cutting-edge artificial intelligence technology and machine learning algorithms, these robots can better understand and adapt to patients’ needs, providing personalized nursing services (46, 108). Additionally, robots will have enhanced autonomous navigation capabilities (109), enabling flexible movement in complex environments and rapid adaptation to changes.
(2) Multimodal interaction and emotional expression: to provide a better care experience, future nursing robots will have diverse interaction methods, including voice recognition, posture sensing, and touch feedback (100). This allows for more natural and seamless communication between patients and nursing robots. Furthermore, nursing robots will possess the ability to express emotions through speech (110), facial expressions, and gestures, fostering a stronger emotional connection with patients.
(3) Multitasking collaboration and teamwork: future nursing robots will be capable of multitasking and collaborating efficiently with other medical devices and robot teams (111). For example, in the operating room, nursing robots can collaborate with surgical robots to provide more precise care support. In nursing teams, nursing robots can seamlessly communicate and cooperate with nurses and doctors, facilitating efficient care processes.
(4) Data sharing and remote monitoring: future nursing robots will have the capability for data sharing and remote monitoring. By connecting to electronic health record systems, nursing robots can real-time record and transmit patients’ health data, allowing healthcare professionals to stay informed about patients’ conditions (112). Additionally, nursing robots can enable remote care and receive guidance from doctors through video monitoring and remote control (29), providing timely care services to patients in remote areas or those unable to be physically present.
(5) Resolution of safety and ethical issues: the future development of nursing robots also requires addressing safety and ethical issues. Technologically, nursing robots need to ensure high levels of safety through safety sensors and control systems, safeguarding both patients and the environment (113). Simultaneously, it is essential to establish relevant laws and regulations, along with ethical guidelines, to clarify the responsibilities and relationships between nursing robots and humans, protecting patient privacy and rights.

**Figure 1:**
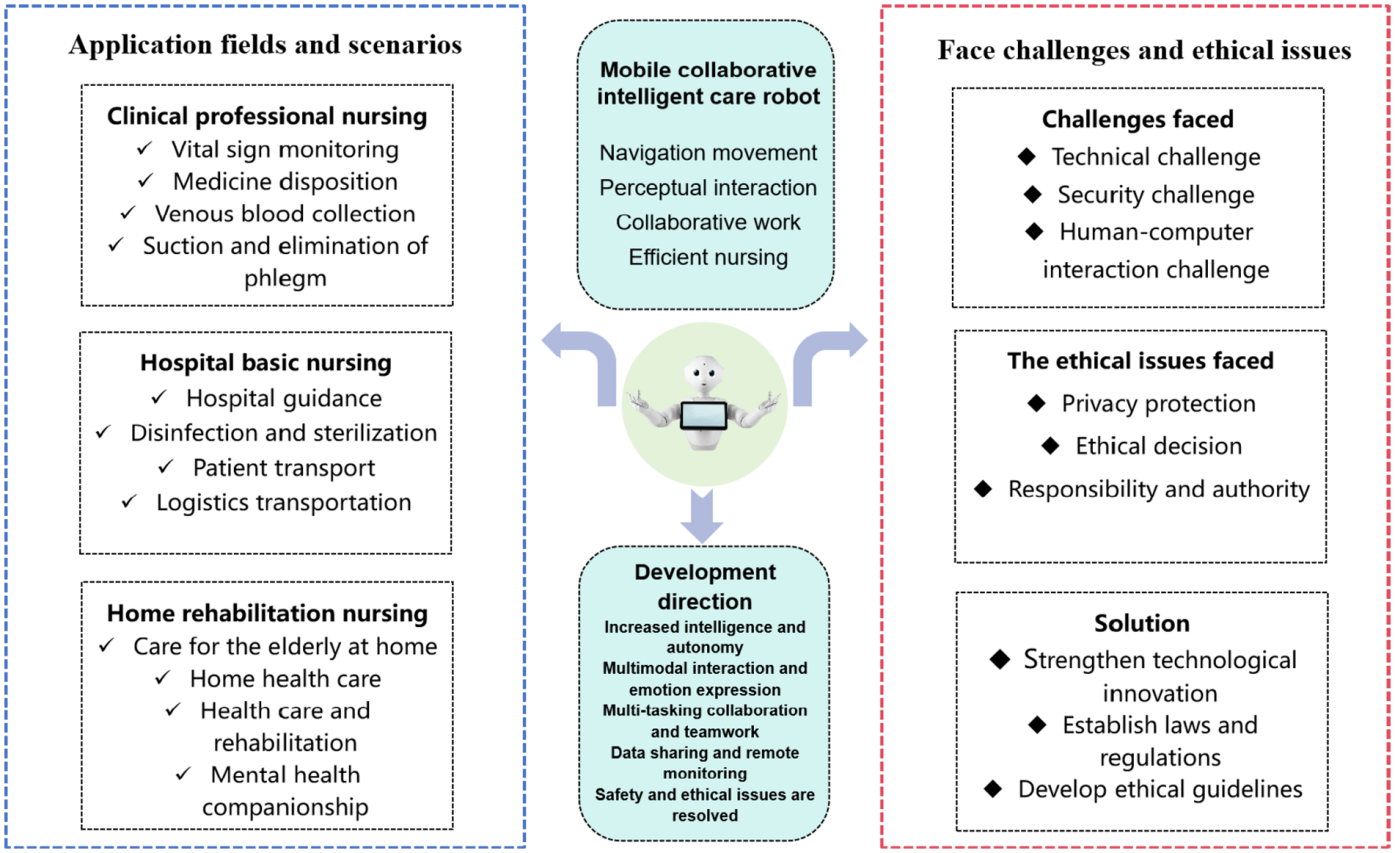
Mobile collaborative intelligent nursing robots article framework summary.

Finally, we summarize the whole review study It is vividly displayed in the form of graphic framework (as shown in Figure 1), which is convenient for readers to understand more intuitively.

## 8 Conclusion

In conclusion, the application of nursing robots spans both clinical settings within hospitals and home care environments outside of medical facilities. Given the distinct differences in application environments and user needs, we chose to discuss these applications in separate chapters. Specifically, clinical applications require robust technological capabilities and adhere to stringent regulatory standards, whereas home care robots focus more on user-friendliness and adaptability to diverse domestic settings. Additionally, there are variances in data management and privacy protection protocols between the two contexts. By delineating these differences through dedicated chapters, we aimed to provide a thorough exploration of the unique challenges and opportunities associated with each setting. However, it is crucial to recognize the interconnectedness of these applications, as advancements in one domain often inform innovations in the other. Both clinical and home care robots contribute to the broader goal of enhancing patient care and improving quality of life, thus forming an integral part of the evolving landscape of healthcare technology.

With the application of technologies such as intelligent enhancement, multimodal interaction, multitasking collaboration, and remote monitoring, the efficiency and convenience of nursing robots in the healthcare field will be further improved. Mobile collaborative nursing robots are expected to become key tools supporting the development of healthcare services. However, it is important to recognize that the development and application of nursing robots still face certain limitations. These include high costs, adaptability in complex environments, and a lack of emotional resonance. Therefore, governments and society need to establish relevant policies to support and regulate the application of nursing robots while protecting patients’ rights and privacy.

Future research on nursing robots should focus on enhancing their perception and cognition capabilities and strengthening their emotional interaction with humans. Additionally, exploring the application of nursing robots in specific areas such as operating rooms and rehabilitation centers, as well as studying their collaboration with other medical devices and personnel, will contribute to improving nursing efficiency. Continuous technological innovation and institutional development will drive the sustainable development of nursing robots and lead to transformative advancements in the healthcare field. Therefore, nursing robots are expected to be more widely applied in the future, leading to improved efficiency and quality of nursing work through close collaboration with doctors, nurses, and other healthcare professionals. This will help alleviate the burden on nursing staff, allowing them to have more time and energy to communicate with and care for patients, providing a more warm and personalized nursing service.
